# Factors Predicting the Quality of Life of University Students in Japan Amidst COVID-19: A Cross-Sectional Study

**DOI:** 10.3389/fpsyg.2022.931381

**Published:** 2022-07-06

**Authors:** Yuko O. Hirano, Risako Uchino, Sae Tanaka, Mutsumi Doi, Koichi Aramaki

**Affiliations:** ^1^Department of Health Sciences, Institute of Biomedical Sciences, Nagasaki University, Nagasaki, Japan; ^2^Department of Health Sciences, Nagasaki University, Nagasaki, Japan; ^3^Graduate School of Biomedical Sciences, Nagasaki University, Nagasaki, Japan

**Keywords:** COVID-19, university students, quality of life, social support, Japan

## Abstract

Owing to the COVID-19 pandemic, classes and club activities in Japanese universities have been canceled; this may increase students' perceived stress and adversely affect their quality of life. This study investigated the factors that influence Japanese university students' quality of life during the pandemic. An online questionnaire collating data related to demographic characteristics, the perceived stress scale (PSS), sense of coherence (SOC), number of social supports, and quality of life (Short Form Health Survey; SF-8) was distributed to university students. Participants were divided into two groups: those who entered university before (seniors) and after (juniors) the onset of the pandemic. Their scores on the two sub-domains (physical and mental) of the SF-8 were evaluated. Multiple regression analysis was performed to identify factors associated with the composite mental summary of the SF-8. Regression analysis indicated that the predictor model of the composite mental summary differed between juniors and seniors. Among seniors, the composite mental summary was significantly indicated by the composite physical summary (β = 0.549, *p* < 0.0001) and PSS (β = 0.422, *p* < 0.0001). Among juniors, it was significantly indicated by the composite physical summary (β = 0.531, *p* < 0.0001), PSS (β = 0.390, *p* < 0.0001), and number of social supports (β = −0.148, *p* = 0.006). The factors associated with quality of life differed between seniors and juniors. Universities must provide opportunities for students to find more friends, especially for juniors who have limited socialization opportunities owing to the pandemic.

## Introduction

The ongoing coronavirus (COVID-19) pandemic is yet to be contained, even 2 years after its outset. Japan banned inbound travel from late January 2020 to control the outbreak of the virus. Travel restrictions led to the cancellation of job fairs for university students (The Japan Times, 2020d) and regular classes after the prime minister called for the closure of schools on February 27, 2020 (The Japan Times, 2020a). A state of emergency (The Japan Times, 2020c), announced on April 7, 2020, followed suit. Therefore, freshmen who entered universities in Japan during the emergency could not attend classes on campus or begin normal university lives. This situation continued even after the partial reopening of grade schools in early May 2020 (The Japan Times, 2020b), considering university students' wide range of activities, which could have further increased the spread of infection. Online classes were offered to university students to compensate for the absence of face-to-face classes. However, these classes can prove physically overwhelming for students, leading to conditions such as eye fatigue (National Federation of University Cooperative, 2020) due to the continuous tasks and assignments.

Moreover, being forced to stay home and restricted from forming close relationships with classmates and peers has affected students' quality of life (QoL). Similar to the reports on university students in Croatia (Dragun et al., 2020), the G20 countries (Nurunnabi et al., 2020), India (Shailaja et al., 2020), Spain (Garvey et al., 2021), and South Korea (Lee et al., 2021), the national and regional lockdowns and travel bans have had a negative influence on the QoL of Japanese university students (National Federation of University Cooperative, 2020). In addition, an international study reported that Japanese students showed more depressive symptoms than Korean and Chinese students (Zhao et al., 2020). This finding indicates that Japanese students' QoL plays a central role in helping them maintain good mental health. However, very few cross-sectional studies have examined the QoL of Japanese students during the COVID-19 pandemic.

Studies from other countries indicated that physical conditions, such as changes in body weight, appetite, and sleep quality, were associated with university students' QoL (Dragun et al., 2020; Shailaja et al., 2020; Garvey et al., 2021). It has also been reported that the degree of QoL varied according to students' university departments of enrollment. In particular, medical students viewed themselves as underutilized and were weaker on the psychological domain of QoL (Chawla et al., 2020; Villanueva et al., 2021). Additionally, gender has been identified as a predictor of students' mental health, influencing their QoL (Babore et al., 2020; Rogowska et al., 2020).

In the normal pre-pandemic period, social support was a significant predictor of university students' QoL (Choi et al., 2019; Kuczynski et al., 2020; Li et al., 2020). However, the COVID-19 pandemic restricted students from socializing with friends and peers (Graupensperger et al., 2020; Liu et al., 2020); this may lead to adverse QoL. However, limited social support may also be negatively associated with a sense of coherence (SOC) (Antonovsly, 1987). In everyday life before the pandemic, SOC was identified as a measure of university students' stress resistance and enabled the maintenance of QoL against perceived stress (Mato and Tsukasaki, 2019; Yano et al., 2019; Nagata et al., 2020).

This study aimed to identify the factors that predict the QoL of Japanese university students spending their university life amidst the COVID-19 pandemic.

## Materials and Methods

### Measures

QoL was measured using the Short-Form Health Survey (SF-8). The SF-8 questionnaire is a shorter version of the SF-36 health survey tool used as a practical measure of health-related QoL (Lefante et al., 2005). This study used the Japanese version of the SF-8, developed by Fukuhara and Suzukamo (2004). The SF-8 comprises eight ordinal items: general health, physical functioning, physical role, bodily pain, vitality, social functioning, mental health, and emotional roles. The SF-8 can be divided into two groups: the first is calculated by adding the scores of general health, physical functioning, physical role, and bodily pain (composite physical summary); the second is calculated by adding the scores of vitality, social functioning, mental health, and emotional roles (composite mental summary). Higher SF-8 scores are associated with more severe adverse QoL. The composite mental summary was taken as an outcome of QoL in this study. Perceived stress, a predictor of the composite mental summary, was measured using the perceived stress scale or PSS (Cohen et al., 1983). Perceived stress was defined in this study as that experienced by respondents in the past month during the pandemic. The Japanese version of the PSS (10 items), developed by Sumi (2006), was used in this study. The SOC comprises three questions on manageability, comprehensibility, and meaningfulness. This study utilized the University of Tokyo Health Sociology version of the SOC scale, also referred to as the SOC-3-UTHS (Togari et al., 2007). Each question is rated on a 7-point scale (1–7). Higher SOC scores are associated with higher stress resistance. Social support was assessed using the Social Support Scale developed by Shima (1992). This scale investigates social support's quantity and quality (emotional and tangible dimensions). A regression model was developed to identify the predictors of the composite mental summary by two participant groups: juniors, who entered the university after the pandemic's onset (April 2020), and seniors who entered the university before the pandemic. In soliciting social support, it can be assumed that it was crucial but difficult for juniors to find and cultivate friendships with peers and classmates at the university, with limited socialization opportunities due to quarantine rules and the cancellation of face-to-face classes and club activities. Therefore, the two groups were used to depict different models that predict the composite mental summary of junior and senior students.

### Eligible Participants

An anonymous online questionnaire was distributed to students at a national university located in the western part of Japan. Participants were selected using the following procedure.

Each student at the target university was provided an e-mail address compatible with the student's ID. Therefore, a tentative list of students with serial ID numbers was made by referring to the number of students published on the university website. Next, stratified random sampling was conducted using the tentative list, and 1,982 students were selected. Informed consent was obtained online from the participants.

### Statistical Analysis

IBM SPSS Statistics Version 25-J was used to calculate descriptive statistics for the participants' general characteristics. Bivariate analysis was conducted by performing chi-square tests and *t*-tests to identify the association between the composite mental summary and the characteristics of each participant. In developing the multiple regression model, Pearson's correlation coefficients were calculated to determine the associations between independent (gender, PSS, composite physical summary, SOC, and the number of social supports) and dependent variables (composite mental summary). Cronbach's alpha confirmed internal consistency.

Multiple regression analysis was conducted to identify the factors associated with mental health-related QoL. The variance inflation factor (VIF) was used to quantify the severity of multicollinearity (1.01 < VIF < 1.22). The level of statistical significance was set at *p* < 0.05.

### Ethical Considerations

Informed consent was obtained from the students online prior to their participation in the survey. Ethics approval for this study was granted by the Biomedical Sciences Ethics Board of Nagasaki University (No. 21092401).

## Results

A total of 1,982 e-mails were sent to the students, of which 314 were answered. Eleven incomplete responses were excluded from the analysis. Thus, responses from 303 students (159 female, 144 male) were analyzed (response rate: 15.3%). Regarding academic department, 223 (73.6%) students were non-medical science majors, and 80 (26.4%) were medical science majors.

The mean PSS score (Range: 3–40) was 20.86 (SD = 6.59). The average number of social supports (Range = 17–60) was 46.22 (SD = 8.81). The average SOC score (Range = 7–21) was 15.5 (SD = 2.9), and Cronbach's alpha was 0.70. The average composite physical summary score was 8.34 (SD = 3.66), with a Cronbach's alpha of 0.78 and a range of 4–22. The average score was 9.39 (SD = 3.76) for the composite mental summary, Cronbach's alpha was 0.76, and the range was 4–20.

### Bivariate Analyses

The correlation coefficients between the independent and dependent variables are presented in Tables 1, 2. Strong correlations were found between the composite mental summary scores and some independent variables, such as PSS and number of social supports. The juniors' scores showed a stronger correlation between composite mental summary and composite physical summary (*r* = 0.668, *p* < 0.001), followed by the PSS (*r* = 0.567, *p* < 0.001), and number of social supports (*r* = −0.205, *p* = 0.005). The composite mental summary scores of the seniors showed a stronger correlation with their composite physical summary scores (*r* = 0.739, *p* < 0.001), followed by the PSS scores (*r* = 0.677, *p* < 0.001), SOC scores (*r* = −0.274, *p* = 0.02), and number of social supports (*r* = −0.238, *p* = 0.009; Tables 1, 2).

**Table 1:** Correlations between the independent and dependent variables (seniors).

	**Gender**	**PSS**	**SOC**	**SS**	**Composite physical summary score**	**Composite mental summary score**
Gender	1					
PSS	0.091	1				
SOC	−0.022	−0.295^**^	1			
Number of social supports	−0.056	−0.284^**^	0.300^**^	1		
Composite physical summary score	0.030	0.423^**^	−0.180^*^	−0.120	1	
Composite mental summary score	0.055	0.677^**^	−0.274^**^	−0.238^**^	0.739^**^	1

*PSS, perceived stress scale; SOC, sense of coherence and SS, number of social supports*.

“**” < 0.01,

“*” < 0.05.

**Table 2:** Correlations between the independent and dependent variables (juniors).

	**Gender**	**PSS**	**SOC**	**SS**	**Composite physical summary score**	**Composite mental summary score**
Gender	1					
PSS	0.041	1				
SOC	−0.101	−0.142	1			
Number of social supports	0.200^**^	0.076	0.367^**^	1		
Composite physical summary score	0.149^*^	0.317^**^	−0.094	−0.076	1	
Composite mental summary score	0.092	0.567^**^	−0.143	−0.205^**^	0.668 ^**^	1

*PSS, perceived stress scale; SOC, sense of coherence and SS, number of social supports*.

“**” < 0.01,

“*” < 0.05.

The characteristics of the respondents by grade group are presented in Table 3. Significant differences were observed between the two groups for age and SOC scores. The results of the Chi-square tests indicated that there were no significant differences between the groups in terms of gender and academic department. The average number of social supports did not differ significantly between junior and senior students. However, when analyzed by subscale, the average score of junior students was considerably higher than that of senior students for the following items: talking to each other (*p* = 0.005), going out together (*p* = 0.008), and teaching each other what they did not understand (*p* = 0.041).

**Table 3:** Characteristics of the participants and comparisons of demographic variables by grade groups.

**Variables**	**Grade groups**	**Mean (SD)**	***p*-value**
Age	Juniors	18.8 (0.89)	<0.001
(Range: 18–25)	Seniors	21.1 (1.01)	
PSS score	Juniors	20.52 (6.07)	0.291
(Range: 3–40)	Seniors	21.37 (7.30)	
Number of social supports	Juniors	46.63 (8.85)	0.316
(Range: 17–60)	Seniors	45.60 (8.75)	
SOC score	Juniors	15.82 (2.73)	0.009
(Range: 7–21)	Seniors	14.94 (3.09)	
Composite physical summary score	Juniors	8.11 (3.55)	0.187
(Range: 4–22)	Seniors	8.68 (3.82)	
Composite mental summary score	Juniors	9.26 (3.52)	0.475
(Range: 4–20)	Seniors	9.60 (4.11)	

*PSS, perceived stress scale; SOC, sense of coherence; SD, standard deviation*.

### Multivariate Analyses

Table 4 shows the determinants of the composite mental summary by grade group. The strongest determinant was the composite physical summary, for both juniors (β = 0.531, *p* < 0.001) and seniors (β = 0.549, *p* < 0.001). The PSS scores, which were positively correlated with the composite mental summary scores in both juniors (β = 0.390, *p* < 0.001) and seniors (β = 0.422, *p* < 0.001), were the next strongest. Furthermore, there was a negative correlation between the composite mental summary scores and the number of social supports (β = −0.148, *p* = 0.006) among the juniors, indicating that the greater the number of social supports, the higher they scored on the composite mental summary. However, such a tendency was not observed among senior students (Table 4).

**Table 4:** Determinants of Composite mental summary scores by grade groups.

	**Juniors**		**Seniors**	
**Items**	**Beta**	***p*-value**	**Beta**	***p*-value**
Gender	0.029	0.564	−0.003	0.953
PSS	0.390	<0.001	0.422	<0.001
Composite physical summary score	0.531	<0.001	0.549	<0.001
SOC	0.019	0.715	−0.038	0.479
Number of social supports	−0.148	0.006	−0.041	0.445
Adj R^2^	0.594	<0.001	0.699	<0.001

*PSS, perceived stress scale; SOC, sense of coherence; Adj R^2^, adjusted R-squared*.

## Discussion

The present study clarified the factors that determined the mental health-related QoL of junior and senior students at a Japanese university. Notably, there was no significant difference in the mean composite mental summary score between the two grade groups.

The composite physical summary and PSS scores were significantly associated with the composite mental summary among senior students. The composite mental summary score was significantly associated with composite physical summary, PSS scores and number of social support among juniors. This finding indicates that social support played a crucial role in determining the composite mental summary for junior students. Therefore, it was hypothesized that social support might act differently in predicting the mental health-related QoL by grade group. Although there were no significant differences between the mean scores of the number of social supports, junior students were more likely to solicit friends to talk to, go out together, and teach each other what they did not understand. This result suggests that even under the restricted socialization conditions due to COVID-19, junior students attempted to solicit social support more often than seniors by maximizing any socialization opportunities.

Senior students had existing social support from peers and friends obtained before the pandemic's onset through regular face-to-face classes and club activities. In other words, in the case of senior students, existing social support was available whenever needed, and therefore, they did not need to acquire new sources of support. However, juniors needed to find social support independently by talking to other students in the classroom or developing the relationship further by going out together and exchanging necessary information to maintain a good QoL. These actions are successful coping strategies to survive the “new normal,” where everyone needs to adjust to daily life amid the pandemic.

According to Antonovsly (1987) theory, maximizing general registrant resources (GRRs) can achieve a successful coping strategy. Social support is one of the types of GRRs and was found to work more efficiently for juniors. The correlation between the number of social supports and SOC score was stronger in juniors than in seniors (Tables 1, 2). Indeed, the SOC scores did not significantly predict the composite mental summary. Nonetheless, it is worth paying attention to the different correlations between the PSS and SOC scores in juniors and seniors. The PSS score was negatively correlated to the SOC score and the number of social supports among senior students. In contrast, the PSS scores of junior students showed a low correlation with their SOC scores and number of social supports. This finding indicates that the stress process model may not apply to junior students.

A few studies have previously indicated the association between academic year and mental health status during the COVID-19 pandemic. Ma et al. (2020) found that the COVID-19 outbreak was significantly associated with acute stress, depression, and anxiety among senior year students. The interpretation of this phenomenon is that first-year students tend to have less academic pressure and anxiety about future employment than seniors (Cao et al., 2020; Sprung and Rogers, 2021). If this is true, senior students in the study may have suppressed perceived stress by using as many coping strategies as possible, such as social support and SOC. In the case of juniors, such coping strategies were not effective in suppressing perceived stress. This result indicates that coping strategies for perceived stress during the pandemic may vary between grade groups. Further studies are needed to probe the association between perceived stress, SOC, and social support for students who began university life after the onset of the COVID-19 pandemic.

This study may be subject to sampling bias. Although the students learned to use information technology in high school and could easily access the online survey with their cellphones and digital devices, it is possible that some students felt anxious about accessing the survey and thus could not participate in the study.

Nonetheless, the implications of this study are crucial for health policies that target university students in Japan, as most universities have no choice but to limit face-to-face lessons amidst the ongoing COVID-19 pandemic. The present findings indicate the need to develop an appropriate program to help students maintain a good QoL in the “new normal” era. As an essential source of social support for students, universities need to establish systems that can provide students the support needed to maintain their QoL. The following are some recommendations.

To maintain good QoL, it is suggested that universities provide students, particularly juniors, opportunities to meet their peers and develop friendships easily. First, this increases the chances of making friends online and creates opportunities for virtual face-to-face relationships. Conducting online group discussions within a class may be a good opportunity for students to exchange their opinions, thereby understanding each other better. Second, when face-to-face lessons begin, class-based activities are expected to increase. Universities should also provide students with helpful information about COVID-19, which may help them avoid distracting information about the uncertainties regarding the pandemic.

Such information and assistance should not be limited to college life, but it should also include the post-graduate period when they expect to be in employment. Currently, the global youth unemployment rate has increased, due to the recession caused by the COVID-19 crisis. Japan is not an exception. The Ministry of Health, Labor and Welfare reported that the employment rate of university graduates has dropped by 2% in fiscal year 2020 (Ministry of Health, Labor and Welfare, 2021), compared to the previous year. Therefore, it is crucial to motivate students to develop the Life Design approach (Fusco et al., 2021) for a future solution.

Moreover, cellphone and internet use (Kim et al., 2011; Bao et al., 2020) is highly prevalent among students. Therefore, these can be used to provide useful information and assistance, even when movement is restricted due to the pandemic. This component should be included as part of the social support system, especially for junior students, for whom social support significantly increases QoL.

It is also suggested to utilize social support from other sources, such as family members. Because social support from family was associated with low levels of depression and PTSD symptoms of the university students (Liu et al., 2020), the support may contribute to maintain a QoL of the students.

According to the Ministry of Education, Culture, Sports, Science, and Technology, as of June 5, 2020, 60% of universities nationwide provided online classes, while 30% offered online and face-to-face classes (Ministry of Education, Culture, Sports, Science and Technology, 2020). A year later, the situation had not changed (Ministry of Education, Culture, Sports, Science and Technology, 2021). Only 36.4% of universities reported holding solely face-to-face classes. The rest were exclusively or partially providing online classes. The World Health Organization (2021) released the “new normal” campaign, considering that the COVID-19 pandemic would continue for some years, and reiterated that people needed to maintain their wellbeing and adjust to life under these conditions for the next few years. Therefore, university students must find ways to cope with the fear of infection and enrich their academic lives to maintain a high QoL by maximizing sources for managing pandemic-related stress.

## Data Availability Statement

The raw data supporting the conclusions of this article will be made available by the corresponding author on reasonable request.

## Ethics Statement

The studies involving human participants were reviewed and approved by Biomedical Sciences Ethics Board of Nagasaki University. The patients/participants provided their written informed consent to participate in this study.

## Author Contributions

RU, ST, and MD developed the questionnaire and conducted the analysis under the instruction of YH and KA. YH wrote the first draft of the manuscript. All authors contributed to the article and approved the submitted version.

## Funding

This study was funded by Management Expenses Grants, Nagasaki University.

## Conflict of Interest

The authors declare that the research was conducted in the absence of any commercial or financial relationships that could be construed as a potential conflict of interest.

## Publisher's Note

All claims expressed in this article are solely those of the authors and do not necessarily represent those of their affiliated organizations, or those of the publisher, the editors and the reviewers. Any product that may be evaluated in this article, or claim that may be made by its manufacturer, is not guaranteed or endorsed by the publisher.
